# Stability of Two Reserve Antibiotics in Elastomeric Pumps: Ceftazidime-Avibactam and Ceftolozane-Tazobactam

**DOI:** 10.3390/antibiotics14100966

**Published:** 2025-09-25

**Authors:** Joana Erdmann, Linh Anna Trúc Vu, Delia Halbeisen, Katharina M. Rentsch

**Affiliations:** 1 Department of Laboratory Medicine, University Hospital Basel, 4031 Basel, Switzerland; joanadamaris.erdmann@usb.ch; 2 Department of Pharmaceutical Sciences, University of Basel, 4001 Basel, Switzerland; delia.halbeisen@usb.ch; 3 Hospital Pharmacy, University Hospital Basel, 4031 Basel, Switzerland; 4 Department of Experimental and Clinical Pharmacology and Pharmacogenomics, University Hospital Tübingen, 72076 Tübingen, Germany; 5 Faculty of Medicine, University of Basel, 4001 Basel, Switzerland

**Keywords:** stability, OPAT, ceftolozane-tazobactam, ceftazidime-avibactam

## Abstract

**Background/Objectives**: Parenteral antibiotic therapy is indispensable in the treatment of several infections. The parenteral administration often leads to the need for prolonged hospitalization. Therefore, interest in outpatient parenteral antimicrobial therapy (OPAT) is growing. OPAT is typically administered in elastomeric devices, which release an infusion solution over a 24 h period and are meanwhile worn close to the body. This work aimed to evaluate the stability of the reserve antibiotics ceftazidime-avibactam and ceftolozane-tazobactam for OPAT use. **Methods**: Elastomeric pumps were prepared in triplicate at the dosages 3.75 and 7.5 g of ceftazidime-avibactam and 2.25, 4.5, and 9.0 g of ceftolozane-tazobactam in 240 mL 0.9% saline each. The pumps were first stored at 2–8 °C for 7 days and subsequently incubated for 48 h at 25 °C. To determine actual concentrations of ceftazidime, avibactam, ceftolozane, tazobactam, and pyridine, samples were taken at nine different time points during storage and incubation. High-performance liquid chromatography coupled to tandem mass spectrometry was used for quantification. **Results**: Although concentrations of ceftazidime and avibactam stayed above 90% during a 24 h incubation period at 25 °C, the pyridine limit of the European Pharmacopeia was already exceeded in the ceftazidime-avibactam pumps after the storage time at 2–8 °C. In the ceftolozane-tazobactam pumps, the ceftolozane concentration was stable for 24 h incubation, but tazobactam concentrations decreased below 90% within 12 h in the two higher dosages. **Conclusions**: Accordingly, stability cannot be guaranteed for both tested preparations and their use for OPAT should be thoroughly considered.

## 1. Introduction

Ceftazidime-avibactam and ceftolozane-tazobactam are reserve antibiotics that are used to treat severe bacterial infections like multidrug-resistant *Pseudomonas aeruginosa* infections [1]. Ceftazidime and ceftolozane have a relatively short half-life [1] and they are beta-lactam antibiotics which are known for their time-dependent efficacy [2]. Due to these properties, parenteral continuous infusion is especially interesting for both preparations for use in hospital and outpatient setting.

Outpatient parenteral antimicrobial therapy (OPAT) showed advantages in multiple programs compared to inpatient antibiotic administration. It showed cost-effectiveness and safety [3,4], a reduced length of hospitalization [4,5], and a lower risk of healthcare-associated infections [6]. In practice, mainly elastomeric devices that are worn close to the body and changed every 24 h are used for OPAT. Observed temperatures in elastomeric devices were up to 32 °C, with the highest temperatures during the night [7]. These conditions require careful investigation of stability, especially for potentially fragile antibiotics like beta-lactams.

Previous analyses of ceftazidime-avibactam stability in elastomeric devices demonstrated that avibactam is more stable than ceftazidime [8,9,10]. Several studies showed ceftazidime instability with a degradation of more than 10% after 24 h of incubation, at 37 °C [8,10,11], at 33 °C [12], and after 14 days storage at 2–8 °C, and subsequent incubation at 32 °C [9] or 25 °C [13]. Degradation below 10% after 24 h could only be observed at room temperature [10,11,14]. The degradation of ceftazidime produces the toxic by-product pyridine [2,9,11,12,15,16]. The acceptable concentration of pyridine in ceftazidime formulations containing sodium carbonate is limited to a maximum of 0.4% pyridine per ceftazidime (*w*/*w*), according to the European Pharmacopoeia (Ph. Eur.) monograph [17]. When pyridine concentrations were determined in previous studies, this limit was always exceeded before the end of the 24 h incubation [9,11,12,16].

Preparations containing ceftolozane-tazobactam in elastomeric devices were reported to be mostly stable. After incubation for 24 h at 37 °C, concentrations were reported once to be stable [18] and once to be below 90% [8]. After storage at 2–8 °C for 8 days and subsequent incubation at 32 °C, the combination was stable for OPAT application if 10% degradation was allowed [19]. At 25 °C, it was reported to be stable for at least 24 h [10,18,20].

The aim of this work was to evaluate whether ceftazidime/avibactam preparations of different concentrations are stable and can comply with the allowed pyridine limits during an OPAT application under mild conditions. We also aimed to confirm the stability of ceftolozane/tazobactam at several common concentrations under mild conditions. To mimic a realistic time between preparation and application, stability was investigated after one week of storage at 2–8 °C. Subsequently, an incubation was performed at 25 °C to mimic mild application conditions with a cooled and insulated elastomeric device.

## 2. Results

**Figure 1:**
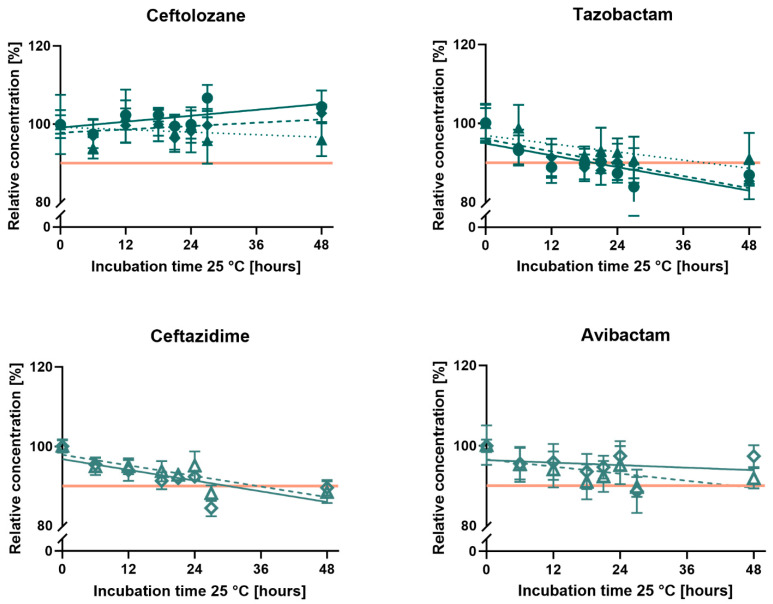
Relative concentrations of ceftazidime, avibactam, ceftolozane, and tazobactam in elastomeric pumps containing 240 mL ceftazidime-avibactam or ceftolozane-tazobactam in 0.9% saline during 48 h of incubation at 25 °C. All pumps were prepared in triplicate at each concentration, and mean concentrations (±standard deviation) of all three pumps were plotted. The horizontal orange line indicates the stability threshold of 90%. The results of the different dosages of ceftolozane + tazobactam are shown with filled triangles and a dashed regression line for 2.25 g (concentration 6.25 + 3.13 g/L), filled rhombuses and a solid regression line for 4.5 g (concentration 12.5 + 6.25 g/L), and filled circles and a dotted regression line for 9.0 g pumps (concentration 25.0 + 12.5 g/L). Results of ceftazidime + avibactam pumps are shown with open triangles and a dashed line for 3.75 g (concentration 12.5 + 3.13 g/L) and open rhombuses and a solid regression line for 7.5 g (concentration 25.0 + 6.25 g/L).

**Figure 2:**
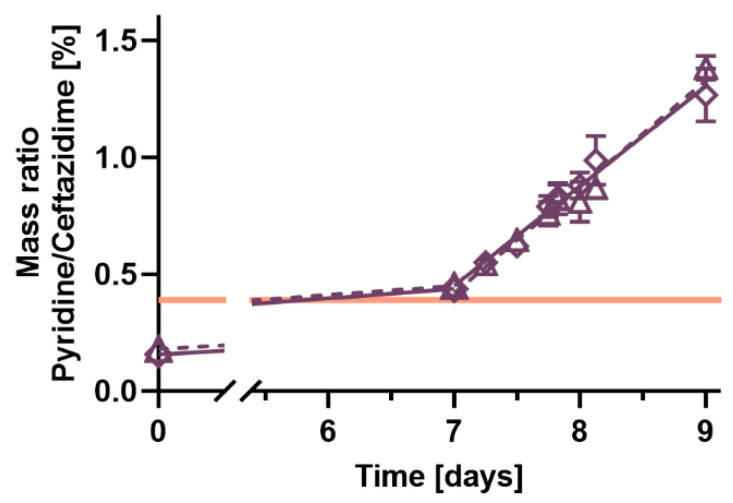
Pyridine/ceftazidime (*w*/*w*) ratios in elastomeric devices containing 240 mL ceftazidime-avibactam in 0.9% saline. Elastomeric devices were first stored at 2–8 °C for 7 days and then incubated at 25 °C for 48 h. The pumps were prepared in triplicate at each concentration, and mean concentrations of all three pumps were plotted. The orange line indicates the upper limit of the pyridine concentration according to Ph. Eur. 10.0 (0.4% of Ceftazidime) [17]. Results of measured pyridine in ceftazidime + avibactam pumps are shown with open triangles and a dashed line for the 3.75 g dosage (concentration 12.5 + 3.13 g/L) and with open rhombuses and a solid regression line for the 7.5 g dosage (concentration 25.0 + 6.25 g/L).

Ceftazidime and avibactam concentrations stayed above 90% for both tested concentrations, 3.75 and 7.5 g ceftazidime-avibactam in 240 mL 0.9% saline, during 24 h of incubation at 25 °C (Figure 1). The toxic degradation product pyridine reached the Ph. Eur. limit of 0.4% pyridine per ceftazidime (*w*/*w*) before the end of the 7-day storage at 2–8 °C, potentially before application to patients (Figure 2). The total pyridine amount formed in the elastomeric pumps after 7 days of storage at 2–8 °C and 24 h incubation at 25 °C was 24 mg pyridine in the elastomeric pumps containing 3.75 g ceftazidime-avibactam and 53 mg pyridine in the 7.5 g ceftazidime-avibactam pumps, respectively.

The elastomeric pumps containing ceftolozane-tazobactam showed stable ceftolozane concentrations above 90% over 24 h at 25 °C, while tazobactam concentrations decreased below 90% in the pumps containing 4.5 g and 9.0 g ceftolozane-tazobactam in 240 mL 0.9% saline. Only the lowest tested concentration of 2.25 g in 240 mL showed concentrations above 90% for both active pharmaceutical ingredients (Figure 1).

The methods for quantification of the different analytes were validated before measuring the samples. Results of the accuracy and precision analysis are shown in Table 1. For all measured analytes, accuracy was within ±12% with a coefficient of variation <13% for all control levels, which was interpreted as sufficient.

## 3. Discussion

Our results indicate that the stability of ceftazidime-avibactam and ceftolozane-tazobactam cannot be guaranteed for OPAT use under the investigated conditions.

The concentration of the toxic degradation product pyridine in elastomeric pumps containing ceftazidime reached the limit of the Ph. Eur. monograph before any significant loss of ceftazidime, which is consistent with previous reports [9,21]. Pyridine, therefore, seems to be the stability-limiting factor in ceftazidime-avibactam OPAT. When comparing the rate of pyridine formation during storage at 2–8 °C and incubation at 25 °C, we observed a higher pyridine formation rate at the higher temperature, which is consistent with published data [9]. Accordingly, shorter incubation time and lower incubation temperature are measures that were recommended to reduce pyridine exposure in continuous infusions of ceftazidime [16]. Compared to Naicker et al., we reduced the storage time from 14 to 7 days and the incubation temperature from 32 °C to 25 °C [9], but pyridine concentrations still did not comply with the Ph. Eur. limit. It is currently discussed whether the exceeded Ph. Eur. monograph threshold for pyridine should limit the use of ceftazidime in OPAT. Jones et al. stated that an OPAT application over 24 h would be safely possible for ceftazidime, even considering the pyridine toxicity [22]. They suggested a new monograph-independent limit with a maximum amount of 100 mg pyridine over a 24 h ceftazidime infusion. This empirical limit was justified by the absence of publications on pyridine toxicity after continuous infusion of ceftazidime. It was also argued that less than 100 mg of pyridine would be produced during a typical continuous infusion with a dose below 6 g/day and a reduced temperature to 15–22 °C. This newly suggested limit would therefore allow a continuous infusion of ceftazidime over 24 h. In our study, the pyridine amount did not exceed the 100 mg limit after storage and incubation for both tested concentrations. Nonetheless, to assure patient safety, we would reinforce the suggestion from Naicker et al. that this newly proposed limit should still be validated with further investigations, like pyridine accumulation in the body [9].

To achieve a reduced pyridine formation in elastomeric devices containing ceftazidime, another approach would be to split the dose and use pumps made for a 12 h application. The shorter application time of infusion, combined with the split dose, was already recommended for continuous infusions of ceftazidime [16]. Further approaches could be to store the prepared devices at −18 °C until application, or to keep the temperature lower than 25 °C during application. It would be important to evaluate the physical stability of the solution and the functionality of the elastomeric device after freezing and thawing. At the same time, it would be important to consider that more frequent changes in the elastomeric devices could also be associated with a higher risk of infection [22].

Our results of the stability analysis of ceftolozane-tazobactam elastomeric devices showed that tazobactam has limited stability. Interestingly, in contrast to our results, in previous reports, tazobactam was more stable than ceftolozane [8,18,19,20], and most tested preparations and conditions were evaluated as stable. Additionally, other formulations containing tazobactam, especially in combination with piperacillin, were reported to be stable and suitable for an OPAT application [7,10,23]. However, we could only observe sufficient stability for the lowest concentration of 2.25 g per 240 mL. Possible explanations for this deviation might be formulation compatibility factors or that the incubation of the devices was performed without light protection. Also, multiple other factors such as pH, diluent, drug concentration, and the elastomeric pump itself, might affect measured concentrations in these infusion solutions [24], leading to a challenging direct comparison to other results. Only one of the previously cited publications had comparable conditions to ours, with the same formulation, the same elastomeric device, the same diluent, and a similar concentration [19]. Whether the decreased tazobactam concentrations, slightly below the threshold of 90% after incubation, would have an impact on the pharmaceutical effect in the patient remain questionable. A pharmacokinetic/pharmacodynamic target attainment analysis and a further toxicological analysis should nonetheless be performed to ensure patient safety.

As a limitation of our study, we did not compare different solvents, storage times, or incubation temperatures. In total, 0.9% saline was chosen as a solvent because both antibiotics were reported to be more stable in saline than in other solvents, like 5% dextrose solutions [8,14]. We tested stability under steady laboratory conditions by storing the elastomeric devices in an incubator with constant temperature. It is known that the temperature in the elastomeric device can vary and reach up to 32 °C during application, if it is worn close to the body, as intended [7]. We suppose that temperatures below 25 °C should be feasible with relatively simple measures like keeping distance between the pumps and the body or using insulated poaches with additional cooling. Another limitation of our study is that no physical stability analysis was performed in addition to the chemical stability measurements. Visual inspection, pH measurement, and particle count are typical analyses that are performed to evaluate the stability of infusion solutions [24]. Assessing physical changes in the solution would deliver additional data about possible incompatibilities. Furthermore, it could help in the explanation of the observed chemical instabilities.

Our stability study of ceftazidime-avibactam in elastomeric pumps delivers new data about pyridine formation in mild conditions, which has not been tested before. Our results confirm the previously published suggestion that mostly the degradation product pyridine limits ceftazidime-avibactam use for OPAT and reinforce the assumption that it is not possible to comply with the Ph. Eur. pyridine limit in an OPAT application. Furthermore, our work delivers additional stability data for ceftolozane-tazobactam in three different concentrations typically used.

Despite the mild incubation conditions, stability requirements were not met for most of the tested preparations. We would therefore recommend careful consideration before an OPAT application. Especially with ceftazidime-avibactam, we would suggest a detailed individual risk–benefit analysis before any potential OPAT application due to the toxic degradation product pyridine.

## 4. Materials and Methods

Elastomeric devices (Easypump II LT 270-27-S from B. Braun Medical AG, Sempach, Switzerland) were filled with a 240 mL infusion solution containing ceftolozane and tazobactam or ceftazidime and avibactam. The elastomeric devices containing ceftolozane-tazobactam were prepared by dissolving Zerbaxa^®^ flasks (Merck Sharp and Dohme AG, Luzern, Switzerland) with 0.9% saline, injecting the corresponding volume into the elastomeric device, and further dilution with 0.9% saline. For the elastomeric devices containing ceftazidime-avibactam, Zavicefta^®^ flasks (Pfizer AG, Zurich, Switzerland) were dissolved with sterile water for injection and further diluted with 0.9% saline. Sterile water for injection was purchased from Fresenius Kabi AG, Kriens Switzerland, and 0.9% Saline was purchased from Laboratorium Dr. G Bichsel AG, Unterseen, Switzerland. Elastomeric devices were prepared in triplicate at each concentration of 2.25, 4.5, and 9 g ceftolozane-tazobactam per 240 mL and of 3.75 and 7.5 g ceftazidime-avibactam per 240 mL, corresponding to standardized daily doses. Each dose describes the sum of beta-lactam and beta-lactamase inhibitor, with a ratio of 2 + 1 for ceftolozane + tazobactam and a ratio of 4 + 1 for ceftazidime + avibactam. The filled elastomeric pumps were stored for 7 days at 2–8 °C and then incubated at 25 °C for 48 h. Samples were taken out of the pumps at nine different time points: after preparation, after storage at 2–8 °C, and after 6, 12, 18, 21, 24, 27, and 48 h of incubation at 25 °C. After collection, the samples were immediately deep-frozen and stored under light protection at −70 °C until measurement.

To determine the concentrations of the active pharmaceutical ingredients, samples from the elastomeric pumps at each time point were diluted in triplicate 1:500 with water. A total of 50 µL of this dilution or of a calibrator or control was added to 30 µL internal standard solution and 200 µL mobile phase A. After mixing, the prepared samples were stored under light protection in a Thermo PAL Autosampler (Thermo Fisher Scientific, Reinach, Switzerland) at 8 °C. 25 µL of each preparation were injected into a high-performance liquid chromatography (HPLC) system coupled to a mass spectrometer (MS).

The HPLC was performed on an UltiMate 3000 HPLC system (Thermo Fisher Scientific, Reinach, Switzerland) using multiple columns: Accucore XL C18 4 µm 150 mm × 4.6 mm, TurboFlow Cyclone 0.5 mm × 50 mm, and TurboFlow Cyclone P 0.5 mm × 50 mm were purchased from Thermo Fisher Scientific, Reinach, Switzerland. Synergi 4 µm Max-RP 80 A 75 mm × 2 mm was obtained from Phenomenex, Basel, Switzerland. HPLC solvents were prepared with acetone (VWR Chemicals, Dietikon, Switzerland), acetonitrile (Thermo Fisher Scientific, Reinach, Switzerland), ammonium acetate, formic acid, methanol, and 2-propanol (all four from Merck KGaA, Darmstadt, Germany) and water which was purified with a PURELAB Chorus 1 Water Purification System (ELGA LabWater, High Wycombe, United Kingdom). Detailed information on the HPLC-methods for the different analytes, the solvents and the used columns is demonstrated in Table 2, Table 3 and Table 4.

Ceftazidime, avibactam, ceftolozane and tazobactam were quantified using a triple quadrupole MS (TSQ Endura, Thermo Fisher Scientific, Reinach, Switzerland), pyridine was analyzed with an Orbitrap MS (Orbitrap Exploris 120, Thermo Fisher Scientific, Reinach, Switzerland). Both MS were equipped with a heated electrospray ionization source with the following source parameters: spray voltage was 3500 V at positive ionization and 1500 V for negative ionization, sheath gas was set at 50 au, and aux gas at 5 au. The ion transfer tube temperature was 300 °C and the vaporizer temperature 400 °C. The MS transitions used for quantification can be found in Table 5. The response ratios of the analyte and the corresponding isotopic internal standard were used for quantification. Mean concentrations of each sampling time point were calculated out of all three pumps. These mean values were used for evaluation.

For validation of the quantification method, intraday accuracy and precision were determined by measuring five quality controls of each level in one run, and interday accuracy and precision by analyzing three quality controls (QCs) of each level in three different runs. Accuracy was calculated using the ratio of the mean measured concentration to the spiked concentration. Precision was determined by dividing the standard deviation by the mean measured concentration. Accuracy and precision were evaluated for each QC level according to the ICH guidelines and accepted if they were within ±15%, except for the LLOQ ± 20% [25].

The analytes for preparation of calibration standards, quality controls, and internal standard were purchased from multiple manufacturers: avibactam sodium, ceftazidime pentahydrate, ceftazidime-d_5,_ and ^15^N_3_-tazobactam sodium were purchased from Toronto Research Chemicals, Toronto, Canada. Pyridine and tazobactam were purchased from Merck KGaA, Darmstadt, Germany, and ^13^C_5_-avibactam sodium from Alsachim, Strasbourg, France, and pyridine-d_5_ from Thermo Fisher Scientific from Reinach, Switzerland.

For quantification of the different compounds, calibrators and quality controls were prepared by spiking water with methanolic stock solutions. Ceftazidime and avibactam were quantified within the same method with a calibration range of 2–60 mg/L for ceftazidime and 1–18 mg/L for avibactam and quality controls that were prepared at four different levels (Table 1). A methanolic solution containing 10 mg/L ceftazidime-d_5_ and 10 mg/L ^13^C_5_-avibactam was used as an internal standard solution. Another method was used for the quantification of ceftolozane and tazobactam with a calibration range of 2–60 mg/L for ceftolozane and 1–30 mg/L for tazobactam. Four controls with different levels over the whole calibration range were prepared (Table 1), and the used internal standard solution consisted of 20 mg/L ceftolozane-d_6_ and 10 mg/L ^15^N_3_-tazobactam in methanol. To quantify pyridine, we used a third method with a calibration range of 5–500 µg/L and five quality controls. The internal standard was 100 µg/L pyridine-d_5_ in methanol.

## Figures and Tables

**Table 1:** Results of accuracy and precision measurements for method validation.

Analyte	Spiked Concentration [mg/L]	Intraday		Interday	
		Accuracy [%]	Coefficient of Variation [%]	Accuracy [%]	Coefficient of Variation [%]
Ceftolozane	2.0	112	5.0	105	12
	5.0	96.6	4.3	96.2	4.4
	21.0	96.5	2.9	97.8	5.0
	52.5	101	1.4	101	2.7
Tazobactam	1.0	97.0	6.9	99.0	2.7
	2.5	95.4	5.2	100	2.4
	10.5	95.8	4.0	97.6	9.8
	26.3	94.0	3.5	105	5.6
Ceftazidime	2.0	92.6	4.0	94.2	3.5
	5.0	93.4	1.2	94.3	0.7
	21.0	96.8	1.2	97.7	0.7
	52.5	97.5	0.6	96.6	1.1
Avibactam	1.0	95.4	4.4	100	6.5
	2.5	99.2	2.5	98.7	2.4
	6.6	100	3.1	101	2.9
	16.5	98.7	3.6	101	4.6
Pyridine	0.005	104	8.6	93.3	12
	0.015	101	9.8	104	7.4
	0.095	97.1	2.7	97.5	0.6
	0.195	94.8	1.9	98.3	4.1
	0.400	98.4	2.1	98.8	1.0

**Table 2:** HPLC gradient for ceftazidime-avibactam quantification. TurboFlow Cyclone P 0.5 mm × 50 mm was used as pre-column and Accucore XL C18 4 µm 150 mm × 4.6 mm as analytical column. A 200 µL loop was loaded with 80% mobile phase A and 20% mobile phase B. During the second step of the gradient, loop, pre-column, and column were connected.

Step	Duration [s]	Loading Pump				Eluting Pump			
		Flow [mL/min]	A	B	C	Flow [mL/min]	A	B	C
1	30	1.5	100	0	0	0.6	95	5	0
2	30	0.3	100	0	0	0.3	95	5	0
3	90	1.5	100 → 0	0 → 100	0	0.6	95 → 50	5 → 50	0
4	90	0.5	0	100	0	0.6	50	50	0
5	60	0.5	0	0	100	0.6	50 → 20	50 → 80	0
6	60	1.5	0	100	0	0.6	20	80	0
7	30	0.5	0	100	0	0.6	0	0	100
8	60	1.5	80	20	0	0.6	95	5	0
9	120	1.5	100	0	0	0.6	95	5	0

Mobile phase A: 10 mM Ammonium acetate in water + 0.1% formic acid. Mobile phase B: 10 mM Ammonium acetate in acetonitrile + methanol (1 + 1, v + v) + 0.1% formic acid. Mobile phase C: Acetone + acetonitrile + isopropanol (1 + 1 + 1, v + v + v).

**Table 3:** HPLC gradient for ceftolozane-tazobactam quantification. TurboFlow Cyclone 0.5 mm × 50 mm was used as pre-column and Synergi 4 µm Max-RP 80 A 75 mm × 2 mm as analytical column. A 200 µL loop was loaded with 70% mobile phase A and 30% mobile phase B. During the second step of the gradient, loop, pre-column, and column were connected.

Step	Duration [s]	Loading Pump				Eluting Pump			
		Flow [mL/min]	A	B	C	Flow [mL/min]	A	B	C
1	30	1.5	100	0	0	0.5	99	1	0
2	30	0.3	100	0	0	0.2	99	1	0
3	60	1.5	100 → 0	0 → 100	0	0.5	99	1	0
4	60	1.5	0	100	0	0.5	99 → 50	1 → 50	0
5	60	0.5	0	0	100	0.5	50	50	0
6	30	0.5	0	100	0	0.5	50	50	0
7	30	0.5	0	100	0	0.5	0	0	100
8	60	1.5	70	30	0	0.5	99	1	0
9	120	0.5	100	0	0	0.5	99	1	0

Mobile phase A: 10 mM Ammonium acetate in water + 0.1% formic acid. Mobile phase B: 10 mM Ammonium acetate in acetonitrile + methanol (1 + 1, v + v) + 0.1% formic acid. Mobile phase C: Acetone + acetonitrile + isopropanol (1 + 1 + 1, v + v + v).

**Table 4:** HPLC gradient for pyridine quantification. TurboFlow Cyclone P 0.5 mm × 50 mm was used as pre-column and Accucore XL C18 4 µm 150 mm × 4.6 mm as analytical column. A loop was loaded with 90% mobile phase A and 10% mobile phase B. During the second step of the gradient, loop, pre-column, and column were connected.

Step	Duration [s]	Loading Pump					Eluting Pump			
		Flow [mL/min]	A	B	C	D	Flow [mL/min]	A	B	C
1	15	1.5	0	0	0	100	0.6	98	2	0
2	30	0.4	98	2	0	0	0.1	98	2	0
3	180	0.5	0	100	0	0	0.6	98 → 10	2 → 90	0
4	105	0.5	0	0	100	0	0.6	10	90	0
5	30	0.5	0	100	0	0	0.6	0	0	100
6	60	1.5	90	10	0	0	0.6	98	2	0
7	120	1.5	0	0	0	100	0.6	98	2	0

Mobile phase A: 5 mM Ammonium acetate in water + 0.1% formic acid. Mobile phase B: 5 mM Ammonium acetate in acetonitrile + methanol (4 + 1, v + v) + 0.1% formic acid. Mobile phase C: Acetone + acetonitrile + isopropanol (1 + 1 + 1, v + v + v); Mobile phase D: 5 mM Ammonium acetate in water.

**Table 5:** MS transitions for the three different methods.

HPLC-MS Method	Analyte	Polarity	Precursor *m*/*z*	Quantifier *m*/*z* Qualifier *m*/*z*	Collision Energy [V]
Ceftazidime-avibactam ^T1^	Ceftazidime	Positive	547	396 277 468	19.7 16.3 11.9
	Ceftazidime-d_5_	Positive	552	396 277 468	19.2 15.5 10.3
	Avibactam	Negative	264	96.0 80.2 97.1	24.1 26.0 18.9
	^13^C_5_-Avibactam	Negative	269	96.0 80.2 97.0	29.7 26.1 19.7
Ceftolozane-tazobactam ^T2^	Ceftolozane	Positive	667	199 397 607	10.3 17.8 14.4
	Ceftolozane-d_6_	Positive	673	205 139 607	33.5 11.1 25.1
	Tazobactam	Positive	301	168 207 283	13.3 14.1 10.3
	^15^N_3_-Tazobactam	Positive	304	208 168 286	13.1 14.5 10.3
Pyridine ^O^	Pyridine	Positive	80.0500	Precursor *m*/*z* in full scan	
	Pyridine-d_5_	Positive	85.0500	Precursor *m*/*z* in full scan	

^T1^ MS: Triple quadrupole TSQ Endura, HPLC method: Table 2; ^T2^ MS: Triple quadrupole TSQ Endura, HPLC method: Table 3; ^O^ MS: Orbitrap Exploris 120, HPLC method: Table 4.

## Data Availability

The raw data supporting the conclusions of this article will be made available by the authors on request.

## Abbreviations

The following abbreviations are used in this manuscript:

OPAT	Outpatient parenteral antimicrobial therapy
Ph. Eur.	European Pharmacopoeia
HPLC	High-performance liquid chromatography
MS	Mass spectrometer
QC	Quality control

## References

[1] van Duin D., Bonomo R.A. (2016). Ceftazidime/Avibactam and Ceftolozane/Tazobactam: Second-generation beta-Lactam/beta-Lactamase Inhibitor Combinations. Clin. Infect. Dis..

[2] Abdul-Aziz M.H., Alffenaar J.C., Bassetti M., Bracht H., Dimopoulos G., Marriott D., Neely M.N., Paiva J.A., Pea F., Sjovall F. (2020). Antimicrobial therapeutic drug monitoring in critically ill adult patients: A Position Paper. Intensive Care Med..

[3] Erba A., Beuret M., Daly M.L., Khanna N., Osthoff M. (2020). OPAT in Switzerland: Single-center experience of a model to treat complicated infections. Infection.

[4] Burch A.R., Ledergerber B., Ringer M., Padrutt M., Reiber C., Mayer F., Zinkernagel A.S., Eberhard N., Kaelin M.B., Hasse B. (2024). Improving antimicrobial treatment in terms of antimicrobial stewardship and health costs by an OPAT service. Infection.

[5] Briquet C., Cornu O., Servais V., Blasson C., Vandeleene B., Yildiz H., Stainier A., Yombie J.C. (2020). Clinical characteristics and outcomes of patients receiving outpatient parenteral antibiotic therapy in a Belgian setting: A single-center pilot study. Acta Clin. Belg..

[6] Chapman A.L.N., Seaton R.A., Cooper M.A., Hedderwick S., Goodall V., Reed C., Sanderson F., Nathwani D., Good B.B.O.P. (2012). Good practice recommendations for outpatient parenteral antimicrobial therapy (OPAT) in adults in the UK: A consensus statement. J. Antimicrob. Chemother..

[7] Voumard R., Van Neyghem N., Cochet C., Gardiol C., Decosterd L., Buclin T., de Valliere S. (2017). Antibiotic stability related to temperature variations in elastomeric pumps used for outpatient parenteral antimicrobial therapy (OPAT). J. Antimicrob. Chemother..

[8] Loeuille G., D’Huart E., Vigneron J., Nisse Y.E., Beiler B., Polo C., Ayari G., Sacrez M., Demore B., Charmillon A. (2022). Stability Studies of 16 Antibiotics for Continuous Infusion in Intensive Care Units and for Performing Outpatient Parenteral Antimicrobial Therapy. Antibiotics.

[9] Naicker S., Roberts J.A., Won H., Wallis S.C., Unwin S., Jamieson C., Hills T., Gilchrist M., Santillo M., Seaton R.A. (2024). Evaluation of the stability of ceftazidime/avibactam in elastomeric infusion devices used for outpatient parenteral antimicrobial therapy utilizing a national stability protocol framework. JAC Antimicrob. Resist..

[10] Fernández-Rubio B., Herrera-Hidalgo L., de Alarcón A., Luque-Márquez R., López-Cortés L.E., Luque S., Gutiérrez-Urbón J.M., Fernández-Polo A., Gutiérrez-Valencia A., Gil-Navarro M.V. (2023). Stability Studies of Antipseudomonal Beta Lactam Agents for Outpatient Therapy. Pharmaceutics.

[11] Viaene E., Chanteux H., Servais H., Mingeot-Leclercq M.P., Tulkens P.M. (2002). Comparative stability studies of antipseudomonal beta-lactams for potential administration through portable elastomeric pumps (home therapy for cystic fibrosis patients) and motor-operated syringes (intensive care units). Antimicrob. Agents Chemother..

[12] Nguyen T., Menten L., Spriet I., Quintens C., Van Schepdael A., Adams E. (2022). Liquid chromatographic method to follow-up ceftazidime and pyridine in portable elastomeric infusion pumps over 24 h. Electrophoresis.

[13] Stendal T.L., Klem W., Tonnesen H.H., Kjonniksen I. (1998). Drug stability and pyridine generation in ceftazidime injection stored in an elastomeric infusion device. Am. J. Health Syst. Pharm..

[14] Walker S.E., Iazzetta J., Law S., Biniecki K. (2010). Stability of commonly used antibiotic solutions in an elastomeric infusion device. Can. J. Hosp. Pharm..

[15] Zajac M., Siwek J., Muszalska I. (1998). The mechanism of ceftazidime degradation in aqueous solutions. Acta Pol. Pharm..

[16] Bourget P., Amin A., Dupont C., Abely M., Desmazes-Dufeu N., Dubus J.C., Jouani B.L., Merlette C., Nove-Josserand R., Pages J. (2014). How To Minimize Toxic Exposure to Pyridine during Continuous Infusion of Ceftazidime in Patients with Cystic Fibrosis?. Antimicrob. Agents Chemother..

[17] (2020). Ceftazidimum pentahydricum et natrii carbonas ad iniectabile, monograph 2344. European Pharmacopoeia, 10.0.

[18] Raby E., Naicker S., Sime F.B., Manning L., Wallis S.C., Pandey S., Roberts J.A. (2020). Ceftolozane-tazobactam in an elastomeric infusion device for ambulatory care: An in vitro stability study. Eur. J. Hosp. Pharm..

[19] Jamieson C., Drummond F., Hills T., Ozolina L., Gilchrist M., Seaton R.A., Santillo M., Wilkinson A.S., Allwood M.C. (2021). Assessment of ceftolozane/tazobactam stability in elastomeric devices and suitability for continuous infusion via outpatient parenteral antimicrobial therapy. JAC Antimicrob. Resist..

[20] Terracciano J., Rhee E.G., Walsh J. (2017). Chemical Stability of Ceftolozane/Tazobactam in Polyvinylchloride Bags and Elastomeric Pumps. Curr. Ther. Res. Clin. Exp..

[21] Jamieson C., Drummond F., Ozolina L., Wilkinson A.S. Stability Testing of Ceftazidime Solutions for Injection in Elastomeric Devices at 12 mg/mL and 25 mg/mL in 0.9% w/v Saline for Safe Use in Outpatient Parenteral Antimicrobial Therapy (OPAT). BSAC OPAT Conference 2019, 11 December 2019. https://e-opat.com/wp-content/uploads/2020/01/OPAT2019-CAZPoster-28Nov.pdf.

[22] Jones T.E., Selby P.R., Mellor C.S., Cheam D.B. (2019). Ceftazidime stability and pyridine toxicity during continuous i.v. infusion. Am. J. Health-Syst. Pharm..

[23] Negrier L., Mena A.M., Dupont C., Gamache P., Zimbril J.O., Abdoune Y., Karrout Y., Odou P., Genay S., Decaudin B. (2024). The Infusion of Piperacillin/Tazobactam with an Elastomeric Device: A Combined 24-H Stability Study and Drug Solution Flow Rate Analysis. Pharmaceuticals.

[24] Esteban-Cartelle B., Vicente-Oliveros N., Menéndez-Conde C.P., Serrano D.R., Martín-Dávila P., Fortún-Abete J., León-Gil L.A., Alvarez-Díaz A. (2022). Antibiotic stability in portable elastomeric infusion devices: A systematic review. Am. J. Health-Syst. Pharm..

[25] Committee for Medicinal Products for Human Use (2022). ICH Guideline M10 on Bioanalytical Method Validation and Study Sample Analysis Step 5.

